# Low Rates of Fibrosis in Eyes Treated with the Port Delivery Platform with Ranibizumab or with Monthly Ranibizumab in the Archway Trial

**DOI:** 10.1016/j.xops.2026.101276

**Published:** 2026-06-08

**Authors:** Usha Chakravarthy, Chui Ming Gemmy Cheung, Giovanni Staurenghi, SriniVas Sadda, Robyn Guymer, Glenn J. Jaffe, Christine A. Curcio, Nancy M. Holekamp, Isabel Bachmeier, Mahnaz Parian Scherb, Steven Blotner, Mel Rabena, Melina Cavichini Cordeiro, Beatriz G. Armendariz, Madeleine S. Kanku, Dominic Heinrich

**Affiliations:** 1 Queen's University of Belfast, Belfast, Northern Ireland, United Kingdom; 2 Singapore Eye Research Institute, Singapore National Eye Centre, Duke-NUS Medical School, National University of Singapore, Singapore; 3 Eye Clinic, Department of Biomedical and Clinical Sciences, Ospedale Luigi Sacco, University of Milan, Milan, Italy; 4 Doheny Eye Institute, University of California, Los Angeles, Pasadena, California; 5 Center for Eye Research Australia, Royal Victorian Eye and Ear Hospital, University of Melbourne (Department of Surgery), Melbourne, Australia; 6 Department of Ophthalmology, Duke University, Durham, North Carolina; 7 University of Alabama at Birmingham, Birmingham, Alabama; 8 F. Hoffmann-La Roche AG, Basel, Switzerland; 9 Genentech, Inc., South San Francisco, California; 10 Roche Pharma AG, Grenzach-Wyhlen, Germany

**Keywords:** Anti-VEGF, Fibrosis, nAMD, Port Delivery Platform with ranibizumab, PDS

## Abstract

**Purpose:**

To evaluate the proportion of eyes with fibrosis in the Archway neovascular age-related macular degeneration trial, which compared efficacy and safety between monthly injections of ranibizumab with treatment via the Port Delivery Platform with ranibizumab (PDS), an intraocular implant providing continuous release of ranibizumab into the vitreous, in eyes that had previously demonstrated a response to anti-VEGF treatment.

**Design:**

Archway (NCT03677934) post hoc analysis.

**Participants:**

Patients received PDS 100 mg/mL with fixed 24-week refill-exchanges (Q24W; n = 248) or monthly intravitreal ranibizumab 0.5 mg injections (n = 167). Patients with subfoveal fibrosis/atrophy on color fundus photography (CFP) were excluded from the trial.

**Methods:**

Masked graders initially assessed CFP images at baseline, week 48, and week 96 for subretinal fibrosis. If grading was uncertain on CFP, spectral-domain OCT (SD-OCT) scans were used to confirm/rule out fibrosis. Two graders assessed images; a senior grader confirmed all cases and arbitrated disagreement. Subretinal hyperreflective material volumes were quantified using deep learning SD-OCT image segmentation.

**Main Outcome Measures:**

Proportion of study eyes with fibrosis at baseline, week 48, and week 96. Mean (95% confidence interval) change from baseline in best-corrected visual acuity up to week 96 by presence/absence of fibrosis.

**Results:**

The proportion of eyes with fibrosis was low in both arms (PDS Q24W, monthly ranibizumab) throughout the trial (baseline: 5.4%, 4.7%; week 48: 7.2%, 4.7%; week 96: 7.7%, 5.4%). In eyes without baseline fibrosis on CFP, 2.4% (5/210) in the PDS Q24W arm and 0.7% (1/141) in the monthly ranibizumab arm developed fibrosis by week 96. Subretinal hyperreflective material volumes were low throughout the trial in both arms. At week 96, eyes with fibrosis gained 3.2 (–2.0, 8.4; n = 17) ETDRS letters in the PDS Q24W arm and lost –9.9 (–30.3, 10.6; n = 8) letters in the monthly ranibizumab arm.

**Conclusions:**

Rates of fibrosis were low with both continuous delivery (PDS) and monthly ranibizumab in VEGF-experienced patients with no fibrosis at baseline based on CFP. The PDS, which removes the need for monthly injections, represents a useful strategy to reduce treatment burden while preventing development of fibrosis and maintaining visual acuity.

**Financial Disclosure(s):**

Proprietary or commercial disclosure may be found in the Footnotes and Disclosures at the end of this article.

Fibrosis, a wound-healing response to neovascularization, results in permanent collagen deposition and scar formation, and contributes to vision loss in neovascular age-related macular degeneration (nAMD) by altering retinal tissue structure.^1^, ^2^, ^3^ Intravitreal anti-VEGF therapies and dual angiopoietin-2/VEGF-A inhibition are current standards of care for nAMD. Despite ongoing treatment, a recent review observed that a significant proportion of eyes treated with anti-VEGF therapies develop fibrosis over time.^3^ A meta-analysis of data from randomized controlled trials and observational studies published up to May 2022 showed that fibrosis was present in 13% of eyes at baseline and 56% of eyes after 5 years of anti-VEGF therapy.^1^ Further, several recent retrospective cohort studies reported that ∼60% to 70% of eyes had fibrosis after 10 years of anti-VEGF therapy.^4^^,^^5^ These findings highlight an unmet need for therapeutic approaches that more effectively reduce the risk of fibrosis development for patients with nAMD.

The Port Delivery Platform with ranibizumab (PDS) is a surgically placed intraocular implant with a refillable reservoir that continuously delivers a customized formulation of ranibizumab to the vitreous, thus reducing treatment burden relative to intravitreal injections.^6^, ^7^, ^8^ The PDS was approved by the US Food and Drug Administration in 2021 for treating adults with nAMD who have previously responded to ≥2 anti-VEGF injections. Approval was based on findings from the phase III Archway trial (NCT03677934), which showed that PDS 100 mg/mL with every-24-week (Q24W) refills was equivalent to monthly intravitreal ranibizumab 0.5 mg for the primary endpoint of adjusted mean change in best-corrected visual acuity (BCVA) from baseline averaged over weeks 36 and 40.^7^ End-of-study results from Archway showed that PDS Q24W maintained vision and anatomic outcomes for up to 96 weeks.^8^

The purpose of the current post hoc analysis was to investigate whether continuous release of anti-VEGF, without the peaks and troughs in drug concentration that would occur after each intravitreal injection, would beneficially affect incident fibrosis in patients enrolled in the Archway trial. We therefore evaluated the presence of fibrosis in eyes of patients treated with PDS Q24W or monthly ranibizumab over 96 weeks. In addition, we measured changes in well-defined subretinal hyperreflective material (SHRM), which has been used as a proxy for fibrosis in many studies.^3^ We used an automated deep learning spectral-domain OCT (SD-OCT) image segmentation algorithm to obtain volumes of SHRM at every visit. We also tested the relationships between outcomes of fibrosis and SHRM with BCVA, which had performed to protocol during the clinical trial.

## Methods

### Archway Study Design and Patients

Archway (NCT03677934) was a phase III, multicenter, randomized, active comparator, open-label, 96-week trial. The full study design and other methodological details have been published previously.^7^^,^^8^ Briefly, patients included were aged ≥50 years with neovascular lesions involving the macula diagnosed in the study eye within 9 months of screening, with ≥3 prior anti-VEGF intravitreal injections within 6 months of screening, and with a documented anatomic and visual response to anti-VEGF treatment for nAMD. Patients with subfoveal fibrosis, based on color fundus photography (CFP), atrophy, or both, based on multimodal imaging, were excluded from trial entry. Other exclusion criteria included previous treatment for nAMD other than anti-VEGFs and any specific concurrent ocular conditions. Images were graded by the Duke Reading Center.

Eyes were randomized 3:2 to PDS 100 mg/mL Q24W or monthly intravitreal ranibizumab 0.5 mg.^7^ Patients in the PDS arm could receive supplemental treatment with intravitreal ranibizumab at the 2 visits before refill-exchange visits (weeks 16, 20, 40, 44, 64, 68, 88, and 92).

The trial adhered to the tenets of the Declaration of Helsinki and was conducted in accordance with International Conference on Harmonisation E6 Guidelines for Good Clinical Practice and applicable local, state, and federal laws. All trial sites received institutional review board approval before trial initiation and all patients provided written informed consent before enrollment. An independent data monitoring committee evaluated safety and study conduct.

### Multimodal Fibrosis Grading

Only study eyes were analyzed for the presence of macular fibrosis. Two graders (M.S.K. and D.H., both board-certified ophthalmologists), for whom treatment was masked, independently evaluated all CFP images at week 96, week 48, and baseline, and a senior grader (N.M.H., board-certified ophthalmologist) confirmed all cases of fibrosis and provided adjudication in any cases of uncertainty or disagreement. Grading started with week 96 images and was performed for week 48 and baseline images for only those eyes with fibrosis confirmed at week 96. Fibrosis was classified on CFP as being (1) present: white, yellow, or dark pigment, with higher opacity than healthy retinal tissue; (2) absent: no signs of fibrosis; or (3) uncertain: presence/absence of fibrosis could not be accurately determined. For CFP images where fibrosis grading was uncertain, SD-OCT was used to confirm or rule out the presence of fibrosis. The presence of fibrosis was indicated by well-demarcated hyperreflective lesions above and/or involving the retinal pigment epithelium, with loss or disruption of the overlying ellipsoid zone. In cases where there were no signs confirming fibrosis on SD-OCT, fibrosis was graded absent. The methods used to evaluate the presence of fibrosis on CFP and SD-OCT were based on a review of the literature.^9^

Outcomes evaluated in this analysis included the presence of fibrosis at baseline, week 48, and week 96 in all eyes, and the presence of fibrosis at week 96 in eyes without fibrosis at baseline. Change from baseline in BCVA through week 96 in eyes with and without fibrosis at week 96 was also evaluated.

Color fundus photography and SD-OCT were performed by trained, reading center–certified study site personnel. For CFP, photographers took a bilateral series at each photographic session. A modified 3-standard field protocol at the 30°–40° was used. The external fundus reflex photograph was taken with the fundus camera, focusing on any lens opacity or on the pupillary margin if no opacity was present. As previously described,^10^ SD-OCT was performed using either Cirrus (512 × 128 Macular Cube; 5-line HD Raster) or Spectralis (dense volume [20° × 20°, 97-section, setting automatic real-time to 16, high resolution] scan macula centered; dense volume [20° × 20°, 49-section, setting automatic real-time to 16, high speed] scan macula centered; 7-line raster [30° × 5°, 7-section, setting automatic real-time to 25, high resolution] scan macula centered).

### Evaluation of SHRM Volume

Subretinal hyperreflective material volumes were evaluated from SD-OCT scans using a previously described automated deep learning image segmentation algorithm.^11^ Briefly, we trained an algorithm to detect undefined and well-defined SHRM using Spectralis SD-OCT scans from the phase II AVENUE trial (NCT02484690) and applied the algorithm to all available Spectralis SD-OCT 49- and 97-line volume scans from Archway (randomization, n = 366; week 96, n = 303). A variety of terms for SHRM with less clearly defined boundaries are used in the literature, such as “undefined,” “ill-defined,” or “poorly defined” SHRM. For the purpose of this analysis, the term “undefined SHRM” is used.

To develop the algorithm, pixel-level annotations were performed by the Liverpool Ophthalmology Reading Center. One to 7 B-scans were annotated across 334 volumes (1499 B-scans) for SHRM, and each B-scan was labeled as showing undefined or well-defined SHRM based on ≤50% or >50% of the SHRM boundary, respectively, being delineable from the neurosensory retina. The U-Net was trained with 1337 annotated scans over 150 epochs. The holdout set segmentation (162 scans) yielded a sensitivity of 0.976 for well-defined and 0.860 for undefined SHRM (specificity: 0.999 and 0.998; Dice scores: 0.839 and 0.711, respectively). Applied to the Archway SD-OCT data set (all patients with Spectralis SD-OCT 49- and 97-line volume scans), SHRM volumes (nL) were determined for undefined and well-defined SHRM segmentation in the ETDRS 3-mm diameter circle. Example segmentations are shown in Figure S1 (available at www.ophthalmologyscience.org).

### Statistical Analysis

The analysis population comprised patients who received ≥1 study treatment according to the assigned treatment. No formal hypothesis testing was performed in these post hoc analyses. Descriptive summary statistics are provided for both continuous variables (n, mean, standard deviation, minimum, maximum, and 95% confidence interval [CI]) and categorical variables (n, percent). SAS software version 9.4 (SAS Institute, Inc) was used for statistical analysis.

## Results

### Patient Flow

**Figure 2:**
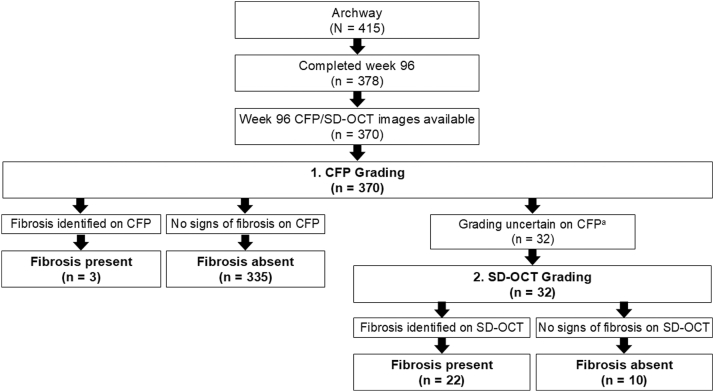
Flow chart summarizing the multimodal approach for grading the presence of fibrosis. Grading started with week 96 images and was only performed for week 48 and baseline images for eyes that had fibrosis confirmed at week 96. A senior grader confirmed all cases of fibrosis and adjudicated disagreement between graders. ^a^Presence/absence of fibrosis could not be accurately determined. CFP = color fundus photography; SD-OCT = spectral-domain OCT.

Overall, 370 Archway eyes had CFP and SD-OCT images available at week 96, which were evaluated as part of this analysis (Fig 2). Among these, CFP confirmed fibrosis in 3 eyes and ruled out fibrosis in 335 eyes. A total of 32 (8.6%) eyes had CFP images where grading was uncertain. OCT grading was therefore performed on these cases, confirming fibrosis in a further 22 eyes and ruling out fibrosis in 10 eyes. Thus at 96 weeks, multimodal defined fibrosis was present in 6.8% (25/370) of eyes.

At baseline and week 48, 4 and 5 eyes, respectively, had CFP images where grading was uncertain; OCT grading subsequently confirmed fibrosis in 4 and 1 of these eyes, respectively.

### Intergrader Reliability

At week 96, the 2 masked graders had near-perfect agreement of >98%. In 7 of the 370 eyes, one or both graders rated the eye as "suspected fibrosis in CFP”; these cases were adjudicated by the senior grader who examined both CFP and OCT images. In these 7 cases, 4 eyes were graded as not having fibrosis and 3 were rated as having fibrosis. At weeks 48 and baseline, masked graders were in 100% agreement.

### Demographic and Baseline Characteristics

Full demographic and baseline ocular characteristics have been described previously.^7^ The characteristics of patients included in this analysis were well balanced between treatment arms, including choroidal neovascularization lesion type, with the majority of eyes in each treatment arm having mixed or type 1 lesions (Table 1). Mean (standard deviation) time since diagnosis of nAMD was 5.9 (10.0) months in the PDS Q24W arm and 5.3 (2.0) months in the monthly ranibizumab arm. Patients had a mean (standard deviation) of 5.0 (2.2) and 5.0 (1.5) prior anti-VEGF injections in the PDS Q24W and monthly ranibizumab arms, respectively.

**Table 1:** Demographic and Baseline Ocular Characteristics

Characteristic	PDS 100 mg/mL Q24W (n = 222)	Intravitreal Ranibizumab 0.5 mg Q4W (n = 148)
Age, yrs		
Mean (SD)	75.1 (8.1)	74.7 (7.6)
Range	51–96	54–87
Sex, no. (%)		
Male	94 (42.3)	60 (40.5)
Baseline BCVA, ETDRS letter score		
Mean (SD)	74.4 (10.7)	76.5 (9.4)
Snellen equivalent	20/32	20/30
Baseline CPT, μm		
Mean (SD)	176.8 (57.1)	179.0 (49.9)
Time since nAMD diagnosis, months		
Mean (SD)	5.9 (10.0)	5.3 (2.0)
Number of prior anti-VEGF injections		
Mean (SD)	5.0 (2.2)	5.0 (1.5)
CNV lesion type∗	n = 211	n = 141
Type 1, no. (%)	64 (30.3)	50 (35.5)
Type 2, no. (%)	18 (8.5)	8 (5.7)
Type 3, no. (%)	7 (3.3)	0
Mixed, no. (%)	122 (57.8)	81 (57.4)
No CNV, no. (%)	0	2 (1.4)

BCVA = best-corrected visual acuity; CNV = choroidal neovascularization; CPT = center point thickness; nAMD = neovascular age-related macular degeneration; PDS = Port Delivery Platform with ranibizumab; Q4W = every 4 wk; Q24W = every 24 wk; SD = standard deviation.

∗ Investigator determined.

### Presence of Fibrosis over Time

**Figure 3:**
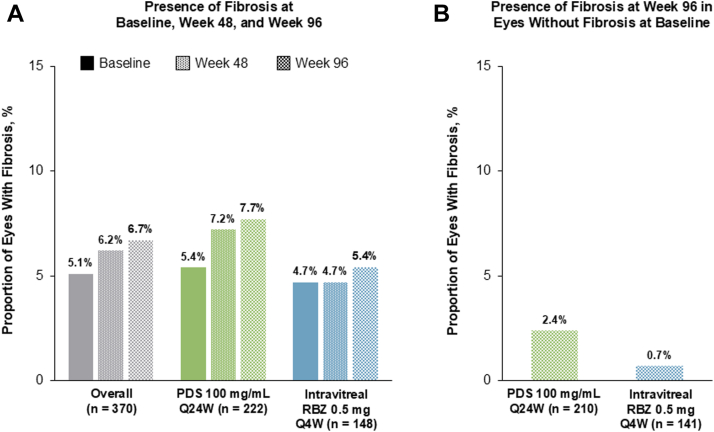
Proportion of eyes (**A**) with fibrosis at baseline, week 48, and week 96, and (**B**) with fibrosis at week 96 in eyes without fibrosis at baseline. PDS = Port Delivery Platform with ranibizumab; Q4W = every 4 weeks; Q24W = every 24 weeks; RBZ = ranibizumab.

The proportion of eyes with fibrosis was low at baseline (5.4% [12/222] of eyes in the PDS Q24W arm and 4.7% [7/148] of eyes in the monthly ranibizumab arm) and through week 96 (7.7% [17/222] of eyes in the PDS Q24W arm and 5.4% [8/148] of eyes in the monthly ranibizumab arm) (Fig 3A).

### Presence of Fibrosis at Week 96 in Eyes without Fibrosis at Baseline

In eyes without fibrosis at baseline, a low proportion developed fibrosis by week 96 (2.4% [5/210] in the PDS Q24W arm and 0.7% [1/141] in the monthly ranibizumab arm) (Fig 3B).

**Figure 4:**
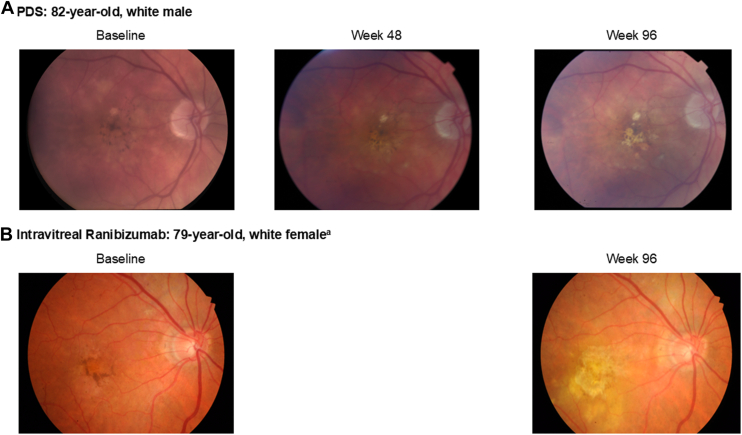
Color fundus photography images for a patient from each arm who developed fibrosis during the trial. (**A**) Port Delivery Platform 100 mg/mL Q24W: 82-year-old, white male. Best-corrected visual acuity: baseline = 72 letters; week 48 = 67 letters; week 96 = 55 letters. Central subfield thickness: baseline = 266 µm; week 48 = 501 µm; week 96 = 363 µm. (**B**) Intravitreal ranibizumab 0.5 mg Q4W: 79-year-old, white female. ^a^Week 48 image not available due to missed study visit. Best-corrected visual acuity: baseline = 78 letters; week 96 = 22 letters. Central subfield thickness: baseline = 350 µm; week 96 = 441 µm. Q4W = every 4 weeks; Q24W = every 24 weeks.

Figure 4 shows CFP images for a patient in each arm who developed fibrosis during the trial.

### BCVA in Eyes with and without Fibrosis at Week 96

**Figure 5:**
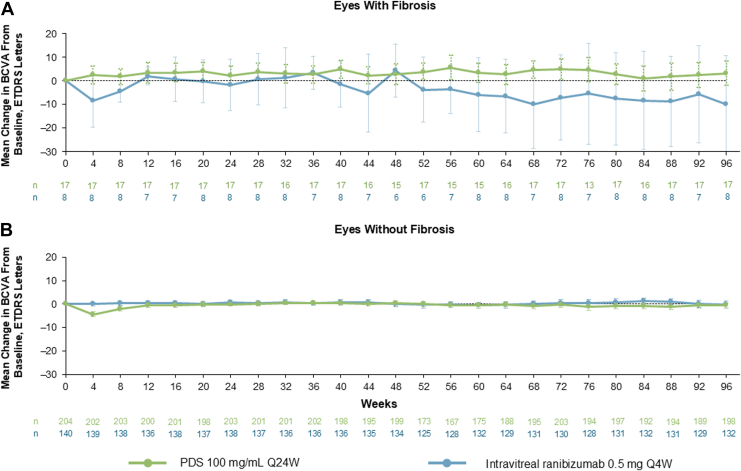
Best-corrected visual acuity change from baseline through week 96 in eyes (**A**) with and (**B**) without fibrosis at week 96. Error bars represent 95% CIs. BCVA = best-corrected visual acuity; CI = confidence interval; PDS = Port Delivery Platform with ranibizumab; Q4W = every 4 weeks; Q24W = every 24 weeks.

Best-corrected visual acuity was maintained in the PDS Q24W arm through week 96, regardless of the presence of fibrosis at week 96 (Fig 5). Mean (95% CI) change from baseline at week 96 was +3.2 (–2.0, 8.4) letters in eyes with fibrosis at week 96 and –0.6 (–1.8, 0.5) letters in eyes without fibrosis at week 96.

Best-corrected visual acuity tended to decrease in the monthly ranibizumab arm through week 96 if fibrosis was present, but the number of patients with fibrosis at week 96 was small (n = 8), and the results showed variability (Fig 5). Mean (95% CI) change from baseline at week 96 was –9.9 (–30.3, 10.6) letters in eyes with fibrosis and –0.2 (–1.6, 1.3) letters in eyes without fibrosis at week 96, although there is insufficient power for a direct statistical comparison of these groups.

### SHRM Volumetric Changes over Time

**Figure 6:**
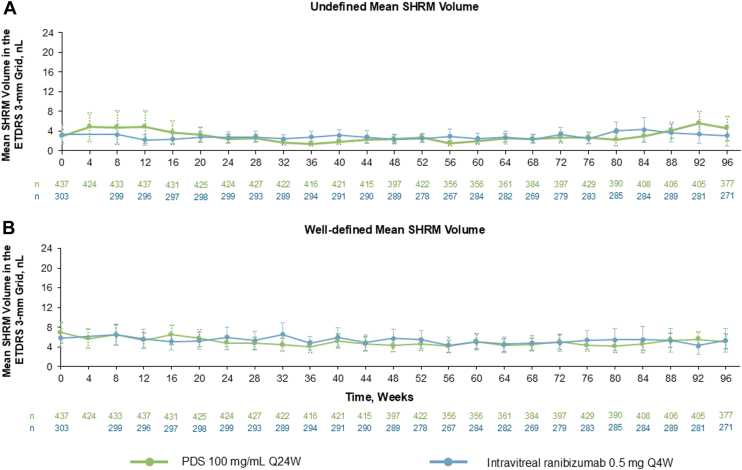
(**A**) Undefined and (**B**) well-defined mean SHRM volumes in the ETDRS 3-mm diameter from baseline through week 96. The analysis included all available Spectralis SD-OCT 49- and 97-line volume scans from Archway (randomization, n = 366; week 96, n = 303). PDS = Port Delivery Platform with ranibizumab; Q4W = every 4 weeks; Q24W = every 24 weeks; SD-OCT = spectral-domain OCT; SHRM = subretinal hyperreflective material.

Most eyes (>60%) had SHRM at baseline (Table S2, available at www.ophthalmologyscience.org). Mean (95% CI) undefined and well-defined SHRM volumes were low at baseline (PDS Q24W: undefined 3.01 [1.72, 4.30] nL, well-defined 6.87 [4.73, 9.00] nL; monthly ranibizumab: undefined 3.30 [1.35, 5.24] nL, well-defined 5.81 [3.87, 7.75] nL) and through week 96 (PDS Q24W: undefined 4.52 [2.06, 6.98] nL, well-defined 5.10 [3.55, 6.65] nL; monthly ranibizumab: undefined 3.08 [0.94, 5.21] nL, well-defined 5.32 [2.89, 7.75] nL), and similar between treatment arms (Fig 6).

## Discussion

This post hoc analysis of the Archway trial demonstrated that both continuous delivery via the PDS, without the burden of monthly injections, and monthly ranibizumab were associated with low rates of fibrosis development in patients with nAMD who had previously received anti-VEGF treatment. Notably, only a small proportion of eyes (<8%) had fibrosis through to week 96 of the trial and few (<2.5%) eyes without fibrosis at baseline developed fibrosis by week 96. This finding suggests that treatment-experienced nAMD eyes without subfoveal fibrosis managed using optimized retreatment posology, such as that achieved by monthly injections and/or continuous release implants without interruptions, are unlikely to develop fibrosis.

Studies of prevalent fibrosis at 2 years from prior nAMD trials that had protocol-mandated monthly assessment and retreatment with anti-VEGF, including CATT,^12^ IVAN,^13^ and HARBOR,^14^ reported rates of around 40%–50%, which is much higher than that found with the prescreened population in the current study. Specifically, CATT,^15^ IVAN,^13^ and HARBOR^16^ all enrolled treatment-naïve patients with nAMD, whereas Archway enrolled patients who had previously received, and demonstrated a response to, anti-VEGF treatment while excluding patients with subfoveal fibrosis (on CFP).^7^ Patients enrolled in Archway had a mean time to diagnosis of ∼6 months and had already received a mean of 5 anti-VEGF injections before baseline.^7^ Furthermore, a relatively small proportion (<10%) of eyes in Archway had type 2 choroidal neovascularization lesions at baseline, a reported risk factor for the development of fibrosis.^3^^,^^12^ Hence, patients in Archway likely had a lower risk of developing fibrosis in this specific maintenance setting, unless under-treatment occurred and active disease returned. Historically, within a given study, the rate of fibrosis increases over time, with eyes initially without fibrosis developing fibrosis during the maintenance period. Refinement in and use of different imaging modalities over time may also be a contributing factor. This was not seen in the current study and may be attributable to continuous delivery by the PDS or because the monthly ranibizumab maintained pharmacologically stable levels of drug.

The presence of undefined SHRM pretreatment is a known risk factor for fibrosis.^3^ However, because all Archway patients were treated previously and the presence of fibrosis was an exclusion criterion, SHRM volumes were low at enrollment and remained mostly unchanged during follow up. Notably the volume of undefined SHRM as determined by this exploratory analysis was extremely low and around twofold lower than well-defined SHRM. Interestingly, even well-defined SHRM volumes reduced over time, suggesting that regions of fibrosis can undergo remodeling shrinkage when nAMD lesion activity is kept under good control, which is consistent with the long durability of the PDS.

In the Archway cohort the PDS Q24W maintained vision and retinal anatomy and was generally well-tolerated.^7^^,^^8^ Through week 40 of the Archway trial, the incidence of ocular adverse events was higher in the PDS Q24W arm compared with the monthly ranibizumab arm.^7^ Endophthalmitis occurred in 1.6% of patients, while serious conjunctival erosion, conjunctival retraction, vitreous hemorrhage, retinal tear, and retinal detachment occurred at low rates of <0.8%. Because there are no other intravitreal devices that have a conduit to the subconjunctival space, we could consider adverse events reported for glaucoma shunt devices, which also have an exterior port opening into the subconjunctival space while the interior communicates with the anterior chamber. A systematic review of glaucoma implants found that events such as tube exposure, hyphema, and choroidal detachments occurred at low rates,^17^ which is in line with the PDS. In addition, a recent systematic review found that rates of conjunctival bleb, vitreous hemorrhage, conjunctival erosion, conjunctival retraction, retinal detachment, and hyphema were similar for PDS versus any ocular implant,^18^ and this may improve over time as clinicians become more experienced with the PDS procedures.

We found that BCVA was maintained in the PDS Q24W arm throughout the trial, regardless of the presence of fibrosis (on CFP) at week 96, whereas BCVA tended to worsen over time in the monthly ranibizumab arm in eyes with fibrosis at week 96. However, as there was only a small number of patients with fibrosis and variation in the BCVA results (particularly in the monthly ranibizumab arm), these vision outcomes should be interpreted with caution. Analyses with a larger sample size of eyes with fibrosis are warranted.

A key strength of the current analysis is the use of a high-quality phase III data set. We also employed 2 approaches in the evaluation of fibrosis (manual grading and assessment of well-defined SHRM volume using a deep learning image segmentation algorithm), with consistent findings. A limitation of our analysis is that because only a small number of study eyes had fibrosis at week 96, no meaningful subgroup analyses could be performed. In addition, not every patient had images at week 96, which we assumed were randomly distributed rather than due to any systematic factor that would have led to a different outcome. It is possible that grading images for weeks 0 and 48 only if fibrosis was present at week 96 could have introduced bias. Not only could rare cases of transient fibrosis at weeks 0 and 48 have been missed (leading to underestimation of the true incidence of fibrosis) but also expectation bias may have led to overestimation of fibrosis presence at weeks 0 and 48 when detected at week 96. However, we do not believe that transient fibrosis has been reported, and it is our view that once fibrosis occurs the changes are permanent. Further, this study was limited to the exploration of 2 treatment options and did not examine other treatment options with average treatment intervals longer than every 4 weeks; further work is needed to determine if these newer strategies might impact the prevalence of fibrosis. Color fundus photography has some limitations as a primary imaging modality, with findings from a CATT analysis suggesting that the use of CFP alone can lead to a misclassification of fibrovascular pigment epithelial detachment as fibrosis.^19^ However, as CFP was supplemented with SD-OCT in cases where grading was uncertain, we believe such misclassification is unlikely and do not believe we have systematically underestimated the presence of fibrosis.

In conclusion, the results of this Archway post hoc analysis indicate that continuous ranibizumab delivery into the vitreous via the PDS could be a useful strategy to control lesion activity and potentially minimize development of fibrosis, while maintaining visual acuity without the burden of monthly injections. Our findings provide some insight into the pathogenesis of fibrosis during anti-VEGF therapy, suggesting that in treatment-experienced patients with no subfoveal fibrosis at baseline, consistent anti-VEGF treatment through 96 weeks can help prevent the development of fibrosis over time. Patients from Archway continue to be followed in the Portal extension trial (NCT03683251), which will allow for evaluation of long-term rates of fibrosis in this population.
