# Supplementary material for: Low Rates of Fibrosis in Eyes Treated with the Port Delivery Platform with Ranibizumab or with Monthly Ranibizumab in the Archway Trial
Source: Ophthalmol Sci. 2026 Jun 8;6(8):101276. doi: 10.1016/j.xops.2026.101276 (PMC13380730; doi:10.1016/j.xops.2026.101276)

**Figure S1.** Example SD-OCT scans for illustration of the deep learning image segmentation of undefined (cyan) and well-defined (magenta) SHRM. The first column shows the original scans without segmentations, the second column shows the B-scan view with segmentation overlay, the third column shows the projection view of the segmentation, and the fourth column shows the corresponding CFP images. Case 1 (top): left eye of a patient with nAMD, exhibiting undefined SHRM at baseline that fully resolves under treatment. The vertical yellow lines (B-scan view) indicate the central 1-mm and 3-mm diameter areas, and the yellow rings (projection view) the central 1-mm, 3-mm, and 6-mm diameter areas of the ETDRS macular grid. The CFP images show pigmentary changes in all visits. Case 2 (bottom): left eye of a patient with nAMD, exhibiting well-defined SHRM at baseline that persists under treatment. The vertical yellow lines (B-scan view) indicate the central 3-mm and 6-mm diameter areas, and the yellow rings (projection view) the central 1-mm, 3-mm, and 6-mm diameter areas of the ETDRS macular grid. The corresponding CFP images show extrafoveal whitish-yellowish material that becomes better demarcated over time. CFP = color fundus photography; ETDRS = Early Treatment Diabetic Retinopathy Study; nAMD = neovascular age-related macular degeneration; SD-OCT = spectral-domain OCT; SHRM = subretinal hyperreflective material.

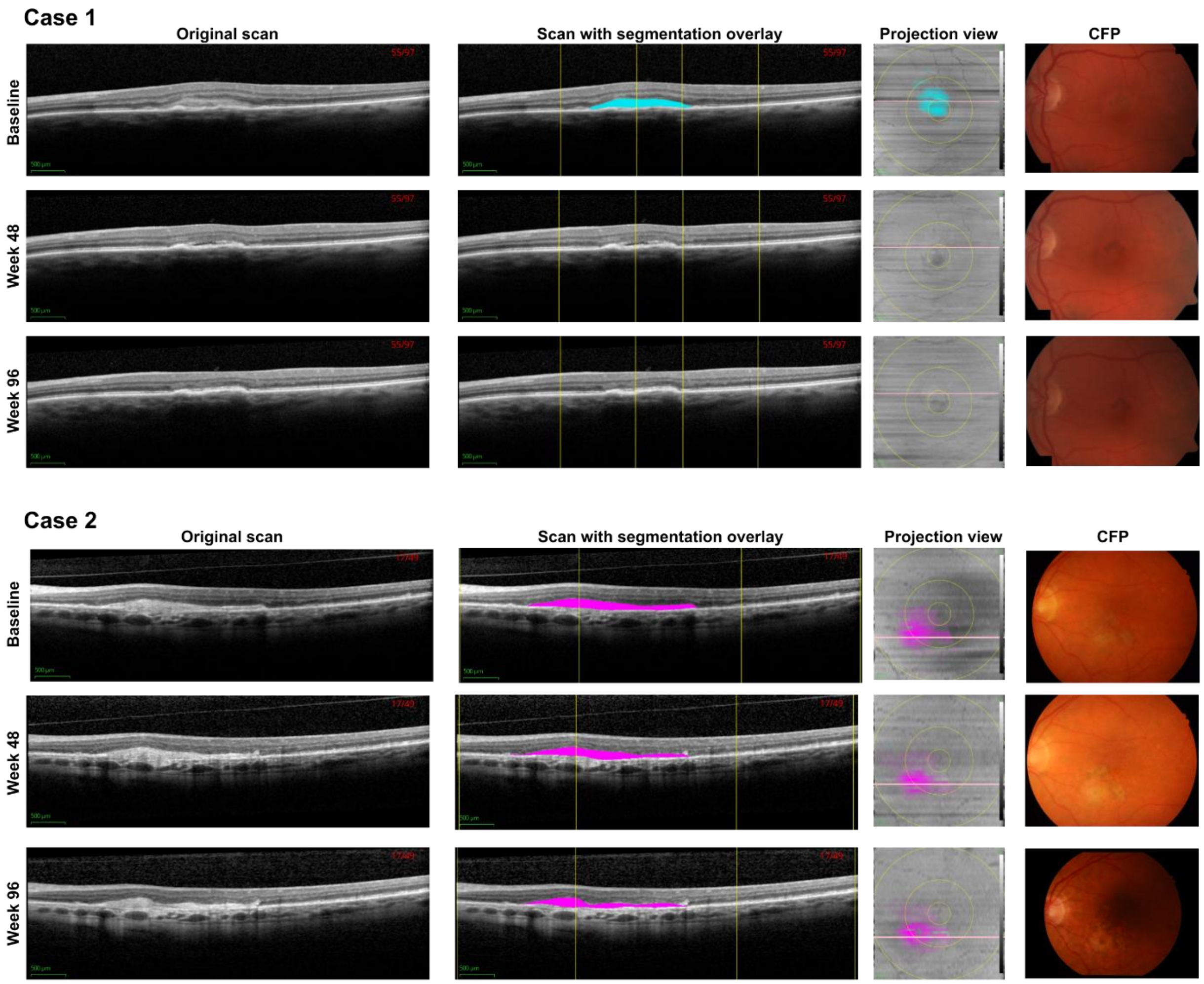

Supplement: Figure S1 [file mmc1.pdf]
