# Supplementary material for: Low Rates of Fibrosis in Eyes Treated with the Port Delivery Platform with Ranibizumab or with Monthly Ranibizumab in the Archway Trial
Source: Ophthalmol Sci. 2026 Jun 8;6(8):101276. doi: 10.1016/j.xops.2026.101276 (PMC13380730; doi:10.1016/j.xops.2026.101276)
Supplement: Table S2 [file mmc2.pdf]

Table S2. Eyes with segmentation of undefined SHRM only, well-defined SHRM only, both undefined and well-defined SHRM, or neither undefined nor well-defined SHRM in the ETDRS 3-mm diameter at baseline.

Segmentation, Eyes, n (%)				
**Neither Undefined Nor Well-Defined SHRM**	**Only Undefined SHRM**	**Only Well-defined SHRM**	**Both Undefined and Well-defined SHRM**	**Total, N (%)**
132 (36.6)	19 (5.3)	132 (36.6)	78 (21.6)	361 (100)

ETDRS = Early Treatment Diabetic Retinopathy Study; SHRM = subretinal hyperreflective material.
